# Sleep Difficulties Are Correlated with Emotional Problems following Loss and Residual Symptoms of Effective Prolonged Grief Disorder Treatment

**DOI:** 10.1155/2013/739804

**Published:** 2013-07-14

**Authors:** Paul A. Boelen, Jaap Lancee

**Affiliations:** ^1^Department of Clinical and Health Psychology, Utrecht University, P.O. Box 80140, 3508 TC Utrecht, The Netherlands; ^2^Department of Clinical Psychology, University of Amsterdam, Weesperplein, 41018 XA Amsterdam, The Netherlands

## Abstract

There is preliminary evidence that poor sleep quality is associated with emotional problems following loss, including symptoms of prolonged grief disorder (PGD) and depression. We conducted two studies to improve existing knowledge about the role of sleep difficulties in recovery from loss. Study 1 that relied on self-reported data from a heterogeneous sample of 177 bereaved individuals replicated prior findings of a linkage between increased sleep difficulties and increased PGD severity. This study also suggested that sleep difficulties are more strongly associated with depression than with PGD. In Study 2, we examined whether prior evidence that sleep complaints are a residual symptom of PGD treatment could be replicated in a sample of 43 bereaved individuals who underwent cognitive behavioural therapy for PGD. Outcomes showed that, although sleep difficulties declined significantly during this cognitive behavioural therapy, after this therapy, over half of all patients still had sleep difficulties above the average sleep difficulties observed in the general Dutch population.

## 1. Introduction

A growing body of empirical studies has shown that an estimated 5 to 10% of people confronted with the death of a loved one develop psychological problems that include generalized anxiety, posttraumatic stress disorder (PTSD), depression, and prolonged grief disorder (PGD) (for reviews see [1, 2]). PGD is characterized by persistent yearning for the deceased, preoccupation with the loss, bitterness, and difficulties to accept the loss [1–3]. A number of empirical studies have shed light on risk factors for PGD and associated disorders following loss; for instance, in a recent review, Burke and Neimeyer [4] conclude that low levels of social support, the loss having a violent cause, being a spouse or parent of the deceased, insecure attachment, and high neuroticism were among the most important prospective predictors of poor bereavement outcome. 

Relatively few studies have yet examined sleep difficulties as a risk factor for postloss psychopathology. Notably, poor sleep quality is a risk factor for different forms of psychopathology, including depression and PTSD [5–7]. A small but growing literature has shown that poor sleep quality is also associated with emotional problems following loss. For instance, several studies have found a positive association between elevated symptom-levels of PGD and more severe sleep disturbances (e.g., [8–10]). Of particular interest is that Germain et al. [11] found that, among bereaved individuals who underwent successful treatment for PGD, poor sleep quality persisted despite clinically significant improvements in PGD. These findings accord with studies showing that sleep disturbances are a frequent residual complaint after successful psychological and pharmacological treatment for depression [12] and PTSD [13]. The finding that sleep disturbances are a residual complaint of successful PGD treatment is important because it implies that sleep disturbances should be addressed in the treatment of PGD to optimize therapeutic gains.

We conducted two studies to improve existing knowledge about the role of sleep difficulties in recovery from loss. In Study 1, self-reported data from a heterogeneous sample of 177 bereaved individuals were used to examine whether prior findings of a linkage between PGD and sleep disturbances could be replicated. Specifically, the first aim of this study was to compare the severity of sleep difficulties between participants who did and did not meet criteria for “caseness” of PGD. In addition, we were concerned with the relative importance of sleep disturbances in symptom-levels of PGD versus depression versus anxiety. There are some studies suggesting that sleep difficulties following loss are more strongly linked to depression than symptoms of disturbed grief [8, 14, 15]. Accordingly, the second aim was to examine correlations between sleep difficulties and symptom-levels of PGD, depression, and anxiety. The third aim was to examine the degree to which sleep difficulties varied as a function of sociodemographic variables (e.g., age, gender) and loss-related variables (e.g., time since loss, mode of death), in order to increase knowledge about bereaved subgroups that are particularly at risk for such difficulties.

In Study 2, we conducted additional analyses of data from 43 patients included in our own treatment trial in which cognitive behavioural therapy successfully alleviated PGD symptoms [16, 17]. The aim of Study 2 was to examine residual sleep disturbances in this group of patients. Based on Germain et al.'s [11] findings of residual sleep disturbances in successfully treated PGD patients, we expected that our cognitive behavioural therapy would improve sleep difficulties but would still leave a considerable number of patients with clinically significant sleep difficulties. 

## 2. Study 1

### 2.1. Materials and Methods

Participants were originally recruited for a research program on cognitive variables in grief, via two sources [18], between the years 2000 and 2004. A first group was recruited via professional and lay mental health-care workers who came in contact with bereaved individuals through their work or voluntary activities; they distributed 1128 questionnaire packets, 492 (43.6%) of which were returned. A second group was recruited through announcements on the internet, inviting bereaved individuals to participate in a survey study either by completing questionnaires online or by completing paper questionnaires sent to their homes. Data for the current study were from 260 participants who chose this latter option. (The digital questionnaire packet was slightly different and was used for other studies within the program.) Of this total group of 752 bereaved individuals (492 recruited from caretakers, plus 260 recruited via internet announcements), 259 had experienced a loss less than one year before inclusion into the research program. This group of 259 relatively recently bereaved individuals was invited to complete additional questionnaires six months after completing the first battery of questionnaires. In total, 177 (68.3%) completed these additional questionnaires. Measures of sleep difficulties were not included in the initial wave of data collection but were included in this additional assessment. Thus, the current study relies on data of 177 bereaved individuals, obtained during this follow-up assessment. Many participants recruited from caretakers likely received some form of psychosocial support between the initial assessment and the follow-up assessment, although we did not register the kind of support they received. However, participants recruited from caretakers and those recruited via internet announcements did not differ in terms of sociodemographic variables or psychopathology measures assessed at follow-up; therefore, it was deemed acceptable to combine these groups of participants for the current study.

Participants had a mean age of 45 (SD = 14.5) years; *n* = 139 (78.5%) were females; *n* = 96 (54.2%) had lost a spouse/partner, *n* = 18 (10.2%) a child, and *n* = 63 (35.6%) some other relative. On average, losses occurred 6.5 (SD = 3.5) months ago and were due to an unnatural cause (i.e., accident, suicide, or homicide) in *n* = 24 (13.6%) cases and a natural cause in *n* = 153 (86.4%) cases. 

 PGD severity was assessed using the Inventory of Complicated Grief (ICG), a measure that includes 19 items (e.g., “Memories of the lost person upset me,” “I feel unable to imagine life being fulfilling without the lost person”) scored on 5-point scales (ranging from 0 = *never*, to 4 = *all the time*). A score of >25 is indicative of PGD “caseness” [19]. Both English [19] and Dutch [20] versions have yielded adequate psychometric properties; although new versions have been developed of the ICG, the original 19-item version and its >25 cut-off score are still frequently used. The *α* in this sample was .84. Sleep difficulties were assessed using the Sleep Difficulties scale from the Dutch version of the Symptom Checklist-90-Revised (SCL-90-R; [21]) originally constructed by Derogatis [22]. This scale instructs respondents to rate the presence of three symptoms (“Trouble falling asleep,” “Waking up early in the morning,” and “Sleep that is restless or disturbed”) during the preceding week, on 5-point scales (1 = *not at all*; 5 = *very much*). In the current study the *α* was .86. We also used the two other scales from the Dutch SCL-90-R, namely, the 16-item Depression Scale (e.g., “Feeling no interest in things,” *α* = .93) and the 10-item Anxiety Scale (e.g., “Feeling fearful,” *α* = .90) to assess symptom-levels of depression and anxiety, respectively.

### 2.2. Results and Discussion

First, we compared the severity of sleep difficulties between participants who did and did not meet criteria for “caseness” of PGD, based on a >25 score on the ICG. In total, *n* = 86 (48.6%) participants were identified as PGD cases and *n* = 91 (51.4%) as noncases. In the PGD group, the average SCL-90-R Sleep Difficulties score was M = 8.6 (SD = 3.9). This score was significantly higher than the mean score of a reference group of 2,361 people from the normal Dutch population (i.e., M = 4.5, SD = 2.2; *t*(85) = 10.06, *P* < .001) obtained from [21]. In the non-PGD group, the average SCL-90-R Sleep Difficulties score was M = 5.9 (SD = 2.9). This score was also significantly higher than the mean score from the normal Dutch reference group (*t*(90) = 4.72, *P* < .001). The percentage of participants scoring above the mean score of the normal reference group (see [21]) was significantly higher in the PGD group (i.e., 70/86 = 81.4%) compared to the non-PGD group (57/91 = 62.64%), Fischer's exact test, *P* = .007. The percentage of participants with minimal sleep difficulties (score of 3 on SCL-90-R Sleep Difficulties scale) was significantly smaller in the PGD group (i.e., 10/86 = 11.6%) compared to the non-PGD group (25/91 = 27.47%), Fischer's exact test, *P* = .009.

Secondly, we examined correlations between sleep difficulties (SCL-90-R) and symptom-levels of PGD (ICG), depression (SCL-90-R), and anxiety (SCL-90-R). The correlation of sleep difficulties with PGD severity was *r* = .43, with depression severity was *r* = .60, and with anxiety severity was *r* = .54 (*ps* < .001). The correlation of sleep difficulties with depression was significantly higher than its correlation with PGD (*Z* = 3.80, *P* < .001) and near-significantly higher compared to its correlation with anxiety (*Z* = 1.69, *P* < .10). The partial correlation of sleep difficulties with PGD (controlling depression and anxiety) was *r*_*p*_ = −.04, *P* = .60; the partial correlation of sleep difficulties with depression (controlling PGD and anxiety) was *r*_*p*_ = .30, *P* < .001; and the partial correlation of sleep difficulties with anxiety (controlling PGD and depression) was *r*_*p*_ = .10, *P* = .19. Taken together, these findings indicate that increased sleep difficulties following loss coincide with elevated symptom-levels of PGD, depression, and anxiety and that sleep difficulties are more strongly a feature of depression than of PGD and anxiety following loss.

Thirdly, we examined the degree to which sleep difficulties varied as a function of several sociodemographic and loss-related variables. For comparative reasons, we also examined associations of these variables with symptom-levels of PGD, depression, and anxiety. Sleep difficulties were positively correlated with age (*r* = .25, *P* < .01) and were more intense among participants who experienced a natural loss versus an unnatural loss (M = 7.5, SD = 3.7 versus M = 5.7, SD = 2.7, *t*(38.4) = 2.87, *P* < .01) but did not vary as a function of the other variables assessed (gender, time since loss, or relationship to the deceased) at a *P* < .05 significance level. Notably, symptom-levels of PGD, depression, and anxiety were unrelated to the sociodemographic and loss-related variables we assessed, at a *P* < .05 level. 

## 3. Study 2

### 3.1. Materials and Methods

Participants were 43 bereaved individuals who underwent a 12-session manualized cognitive behavioural therapy for PGD. They were recruited via self-referral or referral by caretakers and treated at several outpatient clinics in The Netherlands. Participants had suffered a loss at least two months before the start of treatment, met criteria for PGD according to a clinical interview and a >25 score on the ICG (see Study 1), and had PGD as main reason to seek therapy. Exclusion criteria were current substance misuse, psychotic symptoms, severe depression with suicide risk, receiving concurrent psychotherapy, and being illiterate in Dutch (for more details, see [16, 17]). 

Fifty-four patients were included into the study and were allocated to one of three treatment conditions: (i) a cognitive behavioural condition consisting of six sessions of cognitive restructuring followed by six sessions of exposure therapy (*n* = 23); (ii) a cognitive behavioural condition consisting of six sessions of exposure therapy followed by six sessions of cognitive restructuring (*n* = 20); and (iii) 12 sessions of nondirective supportive counselling (*n* = 11). Patients were allocated through a process of minimization stratified on cause of death. Patients completed self-report measures at pretreatment, posttreatment, and six-month follow-up, including the ICG [19, 20] tapping PGD symptoms and the SCL-90-R [21, 22] assessing different domains of psychopathology.

The present analyses were based on scores on the ICG, SCL-90-R Depression scale, and SCL-90-R Sleep Difficulties scale at pretreatment and posttreatment obtained from 43 patients allocated to one of both CBT conditions, that is, conditions (i) or (ii). Participants had a mean age of 43.9 (SD = 13.5) years; *n* = 33 (76.6%) were females; *n* = 11 (25.6%) had lost a spouse/partner, *n* = 9 (20.9%) a child, and *n* = 23 (53.5%) some other relative. On average, losses occurred 37.8 (SD = 44.9) months ago and were due to an unnatural cause (i.e., accident, suicide, or homicide) in *n* = 7 (16.3%) cases and a natural cause in *n* = 36 (83.7%) cases. Internal consistencies of symptom measures at pretreatment and posttreatment, respectively, were *α* = .81 and .85 for the ICG, *α* = .86 and .95 for the SCL-90-R Depression scale, and *α* = .84 and .89 for the SCL-90-R Sleep Difficulties scale. The study was approved by an institutional review board. All participants gave written informed consent. Analyses were conducted using the last observation carried forward method, with pretreatment scores for dropouts included in all analyses.

### 3.2. Results and Discussion

Pretreatment and posttreatment scores on the ICG, SCL-90-R Depression scale, and SCL-90-R Sleep Difficulties scale are shown in Table 1. Paired sample *t*-tests showed that all scores decreased significantly from pretreatment to posttreatment. Sleep difficulties also decreased significantly from pretreatment to posttreatment. However, at posttreatment, 28 of all 43 patients (65.1%) continued to have sleep difficulties above the mean sleep difficulties score found in the reference group of 2,361 people from the normal Dutch population (i.e., M = 4.5; see [21]). At pretreatment this rate was (37/43=) 86%.

Following cognitive behavioural therapy for PGD, 19 (45.2%) patients scored below the cutoff for PGD caseness (i.e., ICG score ≤25). Of these 19 recovered patients, significant sleep difficulties (i.e., sleep difficulties above the mean of the Dutch normal reference group) continued to be endorsed by 10 (52.6%) patients. Of the 24 patients who continued to meet criteria for PGD caseness at posttreatment, significant sleep disturbances continued to be endorsed by 18 (75%) patients. The percentage of patients with significant sleep difficulties at posttreatment did not differ between those who were recovered from PGD (*n* = 19) and those who were not (*n* = 24), Fisher's exact test, *P* = .20. In the recovered group, there were *n* = 3 (15.8%) participants with minimal SCL-90-R Sleep Difficulties (i.e., score of 3) compared to *n* = 4 (16.7%) in the nonrecovered group. These percentages also did not differ, Fisher's exact test, *P* = 1.00.

We calculated residualized change scores for symptom-levels of PGD, depression, and sleep difficulties from pretreatment to posttreatment, representing the changes in these symptom-levels controlling for initial differences and for measurement error inherent in the use of repeated measures with the same instrument [23]. Residualized changes in PGD severity correlated significantly with residualized changes in depression (*r* = .81, *P* < .001) and with residualized changes in sleep difficulties (*r* = .58, *P* < .001). Notably, the association between residualized changes in PGD and depression was significantly stronger than the association between residualized changes in PGD and sleep difficulties (*Z* = 2.36, *P* < .01). Findings of this second study indicate that sleep difficulties decline during cognitive behavioural treatment for PGD but remain a possible problematic residual symptom of remitted PGD.

## 4. Conclusions

The present studies examined the role of sleep difficulties in PGD. Because sleep is a modifiable behaviour, knowledge about its role in PGD and associated symptoms may yield important clues about how to refine therapeutic efforts to facilitate recovery from loss. In Study 1, self-reported data from a heterogeneous sample of bereaved individuals showed that increased sleep difficulties were associated with increased PGD severity. These findings accord with prior studies [8, 9, 11]. In addition, sleep difficulties were significantly more severe among those meeting criteria for PGD caseness compared to those who did not. Notably though, in those not meeting criteria for PGD, almost two-third (57/91 = 62.64%) still had sleep difficulties that exceeded the mean level of sleep difficulties observed in the general Dutch population. This adds to prior research showing that bereavement *per se* interferes with sleep [24, 25]. Sleep difficulties were also associated with symptom-levels of anxiety and depression. Sleep difficulties were more strongly correlated with symptom-levels of depression compared to PGD and anxiety and continued to be associated with depression but not PGD and anxiety, when controlling the shared variance between symptoms. These findings corroborate prior evidence that sleep difficulties following loss are more strongly linked to depression than symptoms of disturbed grief [8, 14, 15] and also add to prior evidence that PGD and bereavement-related depression are distinct phenomena with distinct psychobiological correlates [1, 2]. 

We also examined whether sleep difficulties varied as a function of different sociodemographic and loss-related variables. Outcomes of these analyses showed that sleep difficulties were increased among older bereaved individuals and those confronted with a natural loss in comparison to those confronted with an unnatural loss. The finding of an association between sleep difficulties and age runs counter to prior epidemiological findings [26] and may thus be specific to bereaved individuals. The finding of more sleep difficulties following natural loss seems counterintuitive and contrasts with findings among bereaved college students [9]. That PGD and depression did not differ as a function of sociodemographic and loss-related findings accords with some earlier findings [18] and suggests that other variables (e.g., coping styles) influence these symptoms.

In Study 2 we used data from a prior treatment trial of 43 bereaved individuals with PGD [16, 17] to examine residual sleep difficulties following cognitive behavioural treatment for PGD. Although this treatment coincided with significant declines in PGD, depression, and sleep difficulties, at posttreatment, 65.1% of participants had sleep difficulties above the mean sleep difficulties score found in the general Dutch population [21], compared to 86% at pretreatment. Furthermore, 52.6% of patients who no longer met criteria for PGD caseness at posttreatment continued to have significant sleep difficulties at posttreatment. This rate was not significantly smaller than the 75% who continued to have significant sleep difficulties among those who still met criteria for PGD caseness at posttreatment. Moreover, the recovered and nonrecovered groups did not differ significantly in terms of the number of patients with minimal sleep difficulties at posttreatment. Study 2 also showed that the correlation between declines in PGD severity and declines in sleep difficulties over the course of treatment was significantly smaller than the correlation between declines in PGD severity and declines in depression. The findings of Study 2 accord with findings from Germain et al. [11] who found residual sleep disturbances in PGD patients who were successfully treated for PGD, using “complicated grief treatment”. These findings are also consistent with studies showing that sleep disturbances are a frequent residual complaint after successful treatment for depression [12] and PTSD [13].

The present studies have several limitations. First, data on sleep difficulties were limited to scores on three items from the SCL-90-R (i.e., “Trouble falling asleep,” “Waking up early in the morning,” and “Sleep that is restless or disturbed”). Use of this particular measure allowed us to compare sleep difficulties in our bereaved samples with a large reference group from the general Dutch population [21]. Moreover, there is evidence that using a limited number of sleep items is a valid means to rate sleep difficulties [27]. Yet, using a validated measure for sleep disturbances would have been a more preferable way to examine sleep disturbances in PGD. Secondly and related to this first point, the projects from which these data were drawn were not specifically designed to study sleep difficulties; thus, information was not obtained about, for example, prior history of sleep disturbances in the study samples. Thus, the present findings should be considered provisional until prospectively confirmed in studies specifically designed to investigate sleep difficulties in PGD. 

Notwithstanding these considerations, Study 1 adds to prior evidence of a linkage between PGD and sleep difficulties [8, 9] and prior research suggesting that sleep difficulties are possibly more strongly a feature of bereavement-related depression than symptoms of disturbed grief [14, 15]. Study 2 suggests that although sleep difficulties decline during a cognitive behavioural treatment for PGD, this treatment still leaves a considerable number of patients with sleep difficulties above the average sleep difficulties observed in the general Dutch population. Findings could suggest that sleep difficulties are autonomous symptoms that should be addressed as an integral component of the treatment of PGD or, alternatively, in a follow-up treatment for those patients who successfully recover from PGD yet continue to suffer from significant sleep complaints (cf. [28]). The present findings indicate that cognitive behavioural interventions focused on PGD do not sufficiently reduce sleep difficulties among bereaved individuals and suggest that additional interventions, such as cognitive behavioural interventions focused on insomnia [29, 30], are needed to target such sleep difficulties. Although more studies are needed to corroborate the findings of the studies described in this article, addressing sleep difficulties in the treatment of PGD potentially improves the outcomes of this treatment.

## Figures and Tables

**Table 1:** Means and standard deviations for measures at pre- and posttreatments (Study 2).

	Pretreatment	Posttreatment	*t*	*P*	*d*
	M (SD)	M (SD)			
ICG	42.33 (10.37)	29.47 (13.45)	7.15	<.001	1.07
Depression (SCL-90-R)	48.40 (11.64)	34.42 (14.55)	7.63	<.001	1.06
Sleep difficulties (SCL-90-R)	8.67 (3.50)	6.60 (3.41)	4.17	<.001	0.60

ICG: Inventory of Complicated Grief. SCL-90-R: Symptom Checklist.
